# Psychological Flexibility Is Key for Reducing the Severity and Impact of Fibromyalgia

**DOI:** 10.3390/ijerph18147300

**Published:** 2021-07-08

**Authors:** Miguel A. Vallejo, Laura Vallejo-Slocker, Martin Offenbaecher, Jameson K. Hirsch, Loren L. Toussaint, Niko Kohls, Fuschia Sirois, Javier Rivera

**Affiliations:** 1 Psychology Faculty, Universidad Nacional de Educación a Distancia (UNED), 28040 Madrid, Spain; lauvallejo@madrid.uned.es; 2 Department of Orthopedics, Physical Medicine and Rehabilitation, University Hospital, LMU Munich, 81377 Munich, Germany; martin.offenbaecher@gasteiner-heilstollen.com; 3 Department of Psychology, East Tennessee State University, Johnson City, TN 37614, USA; hirsch@etsu.edu; 4 Department of Psychology, Luther College, Decorah, IA 52101, USA; touslo01@luther.edu; 5 Division of Integrative Health Promotion, University of Applied Science and Arts, 96450 Coburg, Germany; niko.kohls@hs-coburg.de; 6 Department of Psychology, University of Sheffield, Sheffield S1 2LT, UK; f.sirois@sheffield.ac.uk; 7 Rehumatology Unit, Instituto Provincial de Rehabilitación, Hospital General Universitario “Gregorio Marañón”, 28028 Madrid, Spain; javierrivera@ser.es

**Keywords:** fibromyalgia, acceptance, psychological flexibility, catastrophizing, moderation

## Abstract

Fibromyalgia has a significant impact on the lives of patients; symptoms are influenced by psychological factors, such as psychological flexibility and catastrophizing. The objective of this study was to determine the importance of these variables in moderating the association between the severity and impact of fibromyalgia symptoms. A total of 187 patients from a general hospital population were evaluated using the Combined Index of Severity of Fibromyalgia (ICAF), the Fibromyalgia Impact Questionnaire (FIQ), the Acceptance and Action Questionnaire-II (AAQ-II), and the Pain Catastrophizing Scale (PCS). A series of multiple regression analyses were carried out using the PROCESS macro and decision tree analysis. The results show that psychological flexibility modulates the relation between severity and the impact of fibromyalgia symptoms. Catastrophism has residual importance and depends on the interaction with psychological flexibility. Interaction occurs if the severity of the disease is in transition from a mild to a moderate level and accounts for 40.1% of the variance in the sample. These aspects should be considered for evaluation and early intervention in fibromyalgia patients.

## 1. Introduction

Fibromyalgia is a syndrome of unknown etiology characterized by musculoskeletal pain, tiredness, fatigue, and sleep disruption, among other symptoms. It is highly disabling because it alters the lives of patients and carries a high emotional burden. These emotional aspects are part of the syndrome and contribute greatly to its severity. The quality of life of patients is compromised not so much by the pain itself, but by the effect that the syndrome exhibits on their life [1].

The way in which patients cope with the disease is decisive, as it is a chronic disorder, for which, until now, there has only been symptom-focused treatment. According to past research, an array of psychological constructs exert influence on the manifestation and impact of fibromyalgia, including catastrophizing and psychological acceptance or flexibility. Catastrophizing is a tendency to respond to pain from a negative perspective; it mainly has a cognitive component [2], and it is considered an avoidance strategy [3]. Specifically, it is associated with depression in fibromyalgia patients [4].

Acceptance, on the other hand, is considered a way to address the problem by means of psychological flexibility. The presence of discomfort and its limitations are recognized, but personal activities and goals are maintained [5,6]. Thus, acceptance improves functional capacity [7], in addition to improving the emotional state of patients, including catastrophic thoughts [8,9].

The concrete importance of catastrophizing and acceptance of pain, and the interrelation of the two constructs, is complex. Catastrophizing is related to the greater severity of pain, and whereas acceptance is related to less interference in ordinary life; however, there does not seem to be a direct interaction between the two constructs [10]. In the case of fibromyalgia, it has not been observed that these constructs have a mediating effect between pain and the impact of fibromyalgia [4]. However, Vowles et al. [11] found that acceptance mediated the effects of catastrophic thinking on depression and Catala et al. [12] found a moderating effect in the magnification component of catastrophizing between the impact of fibromyalgia and pain.

There is some confusion in determining the clinically relevant effect of these constructs. This confusion is due, in large part, to the selection and conceptualization of the dependent variable. Often, in past research, different dependent variables have been considered simultaneously (e.g., pain intensity, impact of fibromyalgia, depression, and anxiety), with greater impairment, implying greater severity of disease [4,12,13]. Yet, it is important to distinguish between the impact of fibromyalgia, usually measured by the Fibromyalgia Impact Questionnaire (FIQ), and its severity, which cannot be reduced to the impact of the disease or to the intensity of pain or emotional disturbances (e.g., anxiety and depression). A global measure of severity that considers these aspects is more appropriate and allows studying the role of acceptance and catastrophism as potential moderators of the impact-severity linkage.

The Combined Index of Severity of Fibromyalgia (ICAF) is a measure of the severity of fibromyalgia that quantitatively and qualitatively addresses the globality of the disorder, including physical, emotional, and coping aspects [14,15]. The instrument has been shown to be useful in the study of fibromyalgia, making it more suitable for quantifying a global degree of severity [16].

The objective of this study is to examine the importance of catastrophizing and acceptance for determining the severity of fibromyalgia, considering the impact of fibromyalgia, so that it can be known at what gradient of severity such influence occurs. Furthermore, the possible moderating effect of these variables was explored. It is hypothesized that both variables might influence the disease at some point, depending on disease severity, considered together with the impact of the disease on the lives of patients.

## 2. Materials and Methods

### 2.1. Participants

An exploratory study was conducted to evaluate the relations between various disease-related variables and psychological constructs in fibromyalgia patients.

The participants (*N* = 187) were fibromyalgia patients from the Rheumatology Unit of the Rehabilitation Institute (“Gregorio Marañón” Hospital, Madrid, Spain). Patients were invited to participate in the study if they met the following criteria: (a) met the classification criteria for fibromyalgia of the American College of Rheumatology (ACR) 2010 [17,18]; (b) were at least 18 years of age; (c) showed adequate reading comprehension; (d) had a smartphone and were able to use it; and (e) were able to understand and sign the informed consent form. The following were excluded: (a) those with a diagnosis of a mental disorder by a psychiatrist or clinical psychologist; (b) those who had received or were receiving psychological treatment for fibromyalgia or another chronic pain syndrome; and (c) those who had surgery scheduled in the next 3 months.

Sociodemographic data and self-report instruments were obtained through the Qualtrics platform. Data were collected from November 2019 to February 2020.

The Clinical Research Ethics Committee of “Gregorio Marañón Hospital”, Madrid, Spain, approved the study protocol. Informed consent was obtained from each subject. The subjects did not receive any pay or compensation to participate in the research.

### 2.2. Measures

#### 2.2.1. Combined Index of Severity of Fibromyalgia (ICAF)

The ICAF is a questionnaire that quantifies clinical fibromyalgia severity. It consists of 59 items that collect the most common clinical manifestations of fibromyalgia [14]. It provides an overall score; higher scores indicate greater disease severity. It comprises four subscales: emotional, physical activity (pain, fatigue, sleep quality, and functional capacity), active coping, and passive coping. Like the total score, high scores on each subscale indicate greater severity, except for active coping, for which higher scores indicate better coping with the disease. Direct scores are transformed into normalized T scores, with a mean of 50 and a standard deviation of 10 in relation to the studied sample. The weight of each scale in the total score is different. The emotional factor constitutes 66% of the total score, the physical factor constitutes approximately 23%, and the coping factors have relatively little weight, with 6% each. With respect to the total score, a score ranging from 34–41 indicates mild severity, 41–51 is considered moderate severity, and greater than 50 is considered severe [16]. The reliability of the scale is α = 0.85 [14,15]. An example of item is “Please rate your pain by circling the one number that best describes your pain on the average in the past week”.

#### 2.2.2. FIQ

The FIQ [19,20] measures the impact of fibromyalgia on the life of the patient. It consists of 10 items that cover the main areas of life: physical functioning, pain, sleep, mental health, and fatigue. The score ranges from 0 to 100, and higher scores indicate a greater impact of the disease. A score lower than 39 is considered a mild impact, 39 to 59 is considered moderate, and greater than 59 is considered severe [21]. The reliability of the scale is α = 0.82 [20]. An example of item is “Of the 7 days in the past week, how many days did you feel good?”.

#### 2.2.3. Acceptance and Action Questionnaire-II (AAQ-II)

The AAQ-II [22,23] is a self-report measure of psychological flexibility. The lack of will to experience unwanted emotions and thoughts is evaluated, as well as the inability to be in the present moment or use actions directed by values when distressing events are experienced. It is composed of seven items measured on a Likert scale from 1 (never true) to 7 (always true). The total score ranges from 7 to 49, and higher scores indicate high psychological inflexibility. The reliability of the scale is α = 0.88 [23]. An example of item is “Emotions cause problems in my life”.

#### 2.2.4. Pain Catastrophizing Scale (PCS)

The PCS [24,25] is a 13-item scale that assesses pain catastrophism. Participants mark the frequency of 13 negative thoughts or feelings about pain on a Likert scale from 0 (not at all) to 4 (all the time). The total score ranges from 0 to 54. High scores indicate high levels of catastrophic thinking, with respect to pain. The PCS comprises three scales: rumination, magnification, and helplessness. The reliability of the scale is α = 0.79 [25]. An example of an item is “I worry that something serious may happen”.

### 2.3. Data Analysis

The statistical analysis was performed with SPSS version 25. A descriptive analysis was performed for all variables studied. To explore the associations between these variables, a bivariate correlation analysis was conducted.

To determine the influence of the variables studied, a series of multiple regression analyses were conducted using macro PROCESS version 3.5 [26] to determine the influence and possible interaction between the variables. The dependent variable was the severity of fibromyalgia (ICAF), the independent variable was the impact of fibromyalgia (FIQ), and the moderating variables were psychological flexibility (AAQ-II) and catastrophism (PCS), as well as the following interactions: FIQ × AAQ-II, FIQ × PCS, and FIQ × AAQ-II × PCS.

Decision tree analysis was performed using the classification tree module of SPSS 25, with which an optimal algorithm was sought to relate clinical and psychological variables with specific values of fibromyalgia severity (ICAF). This technique uses an algorithm to determine the strongest relationship between the predictors and the outcome variable at each level of the tree. Classification tree analysis is a basic data extraction technique used in different disciplines [27,28]. The dependent variable (criterion) was the ICAF score, and the independent variables were all the other variables studied: the impact of fibromyalgia (FIQ), psychological flexibility (AAQ-II), and the PCS and the three scales that compose it (e.g., rumination, magnification, and helplessness). The chi-squared automatic interaction detector (CHAID) was used. The correction of multiple comparisons was controlled by Bonferroni adjustments. The minimum root and secondary nodes were established at 50 and 25, respectively [29]. The model was validated to evaluate the predictive precision and generalization. A random division of the sample was performed based on the following recommendations [30]: a “training sample” of 70% of the complete sample and a “validation sample” (30%) to test the classification accuracy of the generated decision tree. The data were tested for heteroskedasticity for alpha = 0.05.

The results of the decision tree are more adequate than those provided by the regression analysis. If the relation between the variables is complex and depends on various interactions, the decision tree allows determining these more adequately, highlighting the main ones and not being forced to distinguish between independent, mediating, or moderating variables. In addition, prediction allows the establishment of specific values to make decisions [31].

## 3. Results

### 3.1. Characteristics of the Sample

Table 1 shows the characteristics of the sample. The participants were mostly women (94.7%), and the average age of the participants was 51.6 years. The majority were married (76%) and had a medium education level (45%). More than 50% were active, either as employees (47.3%) or by doing household chores (8.4%). They had been diagnosed with fibromyalgia for a mean of 9.9 years (SD = 4.9). There were no statistical significative differences between sexes in the variables studied. The percentage of female participants in this study is similar to the obtained in a recent prevalence study in Spain [32].

### 3.2. FM Severity, Impact, and Psychological Constructs

Table 2 shows the scores for the participants in the measures used. The mean fibromyalgia severity score (ICAF) was 47.5 (SD = 7.7). The impact of fibromyalgia (FIQ) in patients was high (mean = 64.1; SD = 15). The overall PCS score was 27.4 (SD = 11), and the mean psychological inflexibility score was 28.8 (SD = 10.6). The scores of the questionnaires are similar to other studies. There is only a difference in AAQ-II. Our scores are lower than others obtained in chronic pain [33], and slightly smaller than FM [34]. There are no normative data to ensure the relevance of this difference.

### 3.3. Correlational Analysis

The correlations between the severity of fibromyalgia, its impact, psychological inflexibility, and catastrophizing were all positive and statistically significant, with a large effect size (greater than 0.5). Table 3 provides the values. Only for the correlation between the impact of fibromyalgia and psychological inflexibility was the correlation effect size medium (r = 0.44). There was no significant correlation between the age of the participants and the studied variables or between the duration of the disease and the studied variables, but there was a positive correlation between age and duration of the disease (r = 0.30, *p* < 0.01).

### 3.4. Regression and Moderation Analysis

Three multiple regression models were tested for which the dependent variable was the severity of fibromyalgia and the independent variable was its impact. The first model considers psychological flexibility as a moderating variable, explaining 75% of the variance. A second model explores catastrophism as a moderating variable, explaining 69.7% of the variance. Finally, a third model considers both psychological flexibility and catastrophizing as moderating variables, accounting for 76.1% of the variance. This last model indicates that both interaction effects between psychological flexibility and catastrophizing and the impact of fibromyalgia are significant (F2,181 = 4, *p* = 0.019). As seen in Table 4, the moderating effects of both psychological flexibility and catastrophizing occur in the interaction of these with the independent variable, impact of fibromyalgia, and have a small effect, approximately 1% of the explained variance.

### 3.5. Decision Tree Analysis

**Figure 1:**
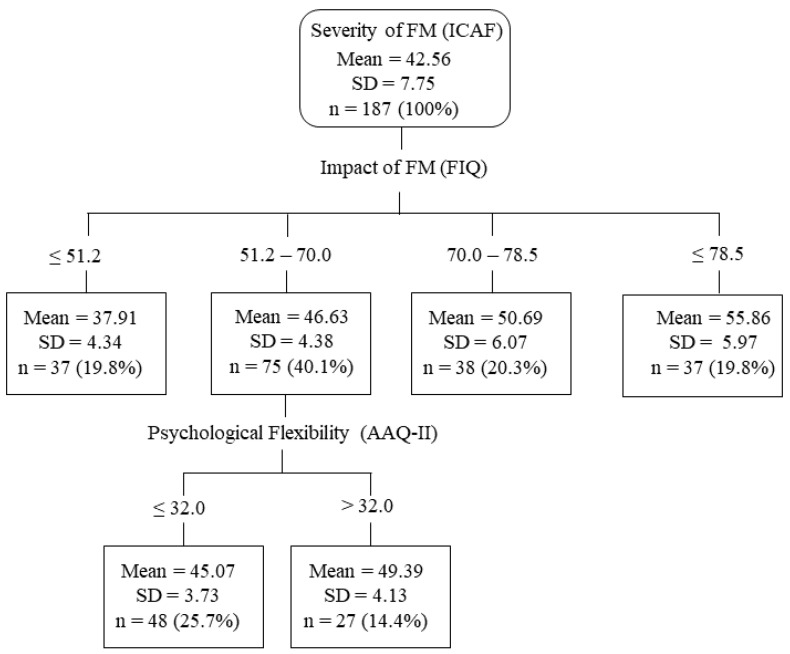
Decision-tree model for severity of FM as the criterion variable.

Considering the severity of fibromyalgia (IACF) as a dependent variable or criterion, decision tree analysis showed a first-level node with the impact of fibromyalgia (FIQ) as a predictor (F3,183 = 82.64, *p* < 0.001). A second-level node, under the fibromyalgia impact node, showed an additional predictor: psychological flexibility (AAQ-II) (F1,73 = 21.42, *p* < 0.001) (Figure 1). No other variable became part of the decision tree. The model was able to correctly classify 88.3% of the sample. The validation of the model showed 90.3% correct classification for the training sample and 89.4% correct classification for the validation sample. The decision tree offers five groups that account for 100% of the sample (19.8, 25.7, 14.4, 20.3, and 19.8).

## 4. Discussion

The severity of fibromyalgia appears to be dependent on the impact of the disease on a patient’s life. Further, the severity of fibromyalgia is not solely determined by pain or fatigue. Emotional variables and modes of coping should be considered, with pain being a relevant element, but not the main one, as was found in other studies [1].

In the current study, we found that psychological flexibility and catastrophizing moderate the relation between the impact of fibromyalgia and its severity. This moderating effect is quantitatively small, but clinically relevant. The decision tree analysis highlights only psychological flexibility and places its influence at moderate levels regarding its impact on fibromyalgia. Thus, when the impact of fibromyalgia is low (less than 51.2 in this study), psychological flexibility may be less relevant. However, when there is a moderate impact of disease (from 51.2 to 70), severity may be slightly reduced or increased; this situation corresponds to 40.1% of the sample, a result that clearly indicates that psychological flexibility helps to mitigate exacerbation of fibromyalgia. Higher values for the impact of the disease do not appear to be influenced by psychological flexibility (see Figure 1). Of note, it is important to compare the results of the decision tree analysis to our regression analyses [35], given the complexity of our study variables and their interrelations.

The results of the regression analysis indicate that the effect obtained from psychological flexibility is similar to that reported by Trainor et al. [36], a small effect, accounting for 2% of the variance and, in our case, even less, approximately 1%. To our knowledge, there are no other published studies that examine the impact of fibromyalgia on severity, and examine the contributions of psychological flexibility and/or catastrophizing. However, other authors have examined the role of catastrophizing and flexibility in the context of pain and other fibromyalgia-related outcomes and have examined disease impact as a predictor rather than outcome. For example, Lami et al. [4] found a significant direct effect for the acceptance of pain and an absence of an effect for catastrophizing, in the prediction of disease impact.

In the treatment of chronic pain, psychological flexibility was shown to be a relevant variable and predictor of better treatment outcomes [37,38,39]. However, catastrophizing has not had this predictive role and is posited to have a more indirect and less causal importance [39]. Indeed, catastrophizing may even exert an influence on psychological inflexibility; for example, in a sample of chronic pain patients, the inclusion of acceptance in the model reduced the impact of catastrophizing on both physical and psychosocial outcomes [11].

The comparison of the results of this work with the findings from other studies is limited by various factors. The first is that the results from samples of individuals with chronic pain and fibromyalgia are not comparable. In studies of chronic pain, the independent variable is usually pain, and the dependent variables are pain interference, depression, anxiety, etc. [40], even when other aspects, such as fatigue, are already being considered [41]. However, fibromyalgia is a syndrome in which pain is not the only or the main symptom. Taking as a reference a more comprehensive measure of the severity of fibromyalgia, as in the case of the ICAF, contributes to more adequately characterizing the object of study.

A second aspect is related to the measurement of psychological flexibility and pain catastrophizing. The AAQ-II is considered a measure of experiential avoidance/acceptance and often as a measure of psychological flexibility. It is a general measure of acceptance [33]. This measure may be more appropriate in the case of fibromyalgia, which, as indicated, is characterized by more than just pain symptoms. Psychological flexibility is broader than symptoms related to pain [42]. The use of specific instruments of psychological flexibility for pain, such as the CPAQ, PIPS, or the explicit reference to pain in the PCS, may not fit adequately with the complaints of fibromyalgia patients [1] or with the inconsistent value of the intensity or the duration of pain and treatment [43,44,45].

It is well known that psychological flexibility is a protective factor [46]. In chronic pain, higher psychological flexibility predicts better improvements in mental health [43]. Recently, Probst et al. [38] reported that internet-based acceptance and commitment therapy was superior to waitlist for participants with low psychological inflexibility at baseline.

This study has several limitations. This cross-sectional correlational study does not allow establishing causal relationships between the variables studied. Our sample size was adequate for a correlational analysis, but could ideally be larger for multivariate analyses performed. The participants were recruited from a specialized department in a general hospital; therefore, they may not be representative of all people with fibromyalgia.

Despite limitations, our study shows the usefulness of evaluating both the severity of fibromyalgia and its impact during patient visits. The possibility of improving psychological flexibility may be important in reducing the severity of fibromyalgia. The transition from mild to moderate fibromyalgia severity is critical [13] because the influence of psychological flexibility occurs at that time [38]; consequently, psychological intervention should occur in that early period of disease progression. An early intervention in FM is a key to prevent a higher severity. The low psychological inflexibility and catastrophizing could be coherent with psychological treatment mainly Acceptance and Commitment Therapy or Cognitive Behavior Therapy. A sort and affordable treatment through Internet could be useful to manage the impact of FM in daily living and reducing pain and fatigue [41]. It should be noted that pain regulation is more efficient with shorter duration of FM [47].

## 5. Conclusions

Both psychological flexibility and catastrophizing play a moderating role between the severity of fibromyalgia and its impact on the lives of patients. For psychological flexibility, this influence occurs when the severity of the disease is in transition between a low and moderate level. Catastrophizing, on the other hand, appears to have less influence. Psychotherapeutic intervention to enhance flexibility and reduce catastrophizing may be an important strategy to help reduce the effects of disease impact on overall severity of fibromyalgia.

## Figures and Tables

**Table 1:** Characteristics of the sample (*N* = 187).

Variable	Percent	*N*	Mean (SD)	Range
Age (y)		187	51.63 (8.13)	28–69
Duration of illness FM (y)		187	9.99 (4.95)	1–16
Sex				
Female	94.70%	177		
Male	5.30%	10		
Marital status				
Single	16.00%	30		
Married	76.50%	143		
Widow	2.10%	4		
Separated, divorced	5.30%	10		
Work status (n)				
Employed	47.30%	108		
Unemployed	24.20%	55		
Homemaker	8.40%	19		
On leave	9.60%	22		
Retired	10.50%	24		
Educational status (n)				
Low (middle school)	19.80%	37		
Medium (high school)	45.50%	85		
High (college)	34.80%	65		

**Table 2:** Mean score (range; SD) (*N* = 187).

Questionnaire	Scores
ICAF	47.55 (29.97–65.41; 7.75)
FIQ	64.08 (23–90; 15.00)
Catastrophizing	27.41 (1–52; 11.02)
Rumination	9.02 (0–16; 3.78)
Magnification	5.65 (0–12; 2.79)
Helplessness	12.74 (0–24; 5.19)
AAQ-II	28.82 (7–49; 10.64)

ICAF: Combined Index of Severity of Fibromyalgia; FIQ: Fibromyalgia Impact Questionnaire; AAQ-II: Acceptance and Action Questionnaire-II.

**Table 3:** Correlations between FM variables and psychological constructs (*N* = 187).

	1	2	3	4	5	6	7
1. ICAF	−−						
2. FIQ	0.77 **	−−					
3. Catastrophizing	0.67 **	0.44 **	−−				
4. Rumination	0.70 **	0.60 **	0.64 **	−−			
5. Magnification	0.60 **	0.54 **	0.55 **	0.94 **	−−		
6. Helplessness	0.62 **	0.48 **	0.57 **	0.89 **	0.80 **	−−	
7. AAQ-II	0.72 **	0.60 **	0.65 **	0.96 **	0.84 **	0.78 **	−−

** *p* < 0.01; ICAF: Combined Index of Severity of Fibromyalgia; FIQ: Fibromyalgia Impact Questionnaire; AAQ-II: Acceptance and Action Questionnaire-II.

**Table 4:** Prospective prediction of severity of FM from impact of FM with several moderator variables (psychological flexibility, catastrophizing, and their interaction). (*N* = 187).

Variable	∆R^2^	R^2^	F	*p*	Beta	t	*p*	95% CI
Severity of FM								
(M1) DV = ICAF	0.01 *	0.74	170.21	<0.001				
FIQ					0.18	3.57	<0.001	0.28, 0.81
AAQ-II					0.08	0.55	0.581	−0.36, 0.20
Interaction					0.004	2.68	<0.001	0.001, 0.008
(M2) DV = ICAF	0.007 *	0.69	138.25	<0.001				
FIQ					0.18	3.67	<0.001	0.28, 0.81
Catastrophizing					0.02	0.16	0.872	−0.29, 0.25
Interaction					0.004	2.13	0.034	0.003, 0.007
(M3) DV = ICAF	0.019 *	0.75	111.42	<0.001				
FIQ (1)					0.13	2.59	0.012	0.03, 0.23
AAQ-II (2)					0.08	0.46	0.642	−0.44, 0.27
Catastrophizing (3)					0.05	0.31	0.751	−0.27, 0.38
Interaction 1 × 2					0.003	1.73	0.084	−0.001, 0.008
Interaction 1 × 3					0.006	0.52	0.059	−0.005, 0.018

* *p* < 0.05, M1: Model 1; M2: Model 2; M3: Model 3; DV: dependent variable; ICAF: Combined Index of Severity of Fibromyalgia, FIQ: Fibromyalgia Impact Questionnaire; AAQ-II: Acceptance and Action Questionnaire-II.

## Data Availability

The study did not report any data.
